# Lung structure and function similarities between primary ciliary dyskinesia and mild cystic fibrosis: a pilot study

**DOI:** 10.1186/s13052-017-0351-2

**Published:** 2017-04-12

**Authors:** Marco Maglione, Silvia Montella, Carmine Mollica, Vincenzo Carnovale, Paola Iacotucci, Fabiola De Gregorio, Antonella Tosco, Mariarosaria Cervasio, Valeria Raia, Francesca Santamaria

**Affiliations:** 1 Department of Translational Medical Sciences, Section of Pediatrics, Federico II University, Via Pansini 5, 80131 Naples, Italy; 2 Biostructure and Bioimaging Institute, National Research Council, Naples, Italy; 3 Department of Translational Medical Sciences, Adult Cystic Fibrosis Center, Federico II University, Naples, Italy; 4 Department of Advanced Biomedical Sciences, Anatomo-Pathology Unit, Federico II University, Naples, Italy

**Keywords:** Computed tomography, Cystic fibrosis, Lung function, Magnetic resonance imaging, Primary ciliary dyskinesia

## Abstract

**Background:**

Primary ciliary dyskinesia (PCD) and cystic fibrosis (CF) are increasingly compared. There are no chest magnetic resonance imaging (MRI) comparative studies of PCD and CF. We assessed clinical, functional, microbiological and MRI findings in PCD and mild CF patients in order to evaluate different expression of lung disease.

**Methods:**

Twenty PCD (15.1 years) and 20 CF subjects with mild respiratory impairment (16 years, 70% with pancreatic insufficiency) underwent MRI, spirometry, and sputum cultures when clinically stable. MRI was scored using the modified Helbich system.

**Results:**

PCD was diagnosed later than CF (9.9 *versus* 0.6 years, *p* = 0.03), despite earlier symptoms (0.1 *versus* 0.6 years, *p* = 0.02). In the year preceding the study, patients from both groups underwent two systemic antibiotic courses (*p* = 0.48). MRI total scores were 11.6 ± 0.7 and 9.1 ± 1 in PCD and CF, respectively. FEV_1_ and FVC Z-scores were −1.75 (range, −4.6–0.7) and −0.6 (−3.9–1.8) in PCD, and −0.9 (range, −5.4–2.3) and −0.3 (−3.4–2.5) in CF, respectively. No difference was found between lung function or structure, despite a higher MRI subscore of collapse/consolidation in PCD *versus* CF (1.6 ± 0.1 and 0.6 ± 0.2, *p* < 0.001). These findings were confirmed after data-control for diagnostic delay. *Pseudomonas aeruginosa* and *Staphylococcus aureus* were more frequent in CF than in PCD (*p* = 0.05 and *p* = 0.003, respectively).

**Conclusions:**

MRI is a valuable radiation-free tool for comparative PCD and CF lung disease assessment. Patients with PCD may exhibit similar MRI and lung function changes as CF subjects with mild pulmonary disease. Delay in PCD diagnosis is unlikely the only determinant of similarities.

**Electronic supplementary material:**

The online version of this article (doi:10.1186/s13052-017-0351-2) contains supplementary material, which is available to authorized users.

## Background

Primary ciliary dyskinesia (PCD) is characterized by altered ciliary beat frequency and/or pattern resulting in impaired airways clearance [1]. Clinical manifestations include neonatal respiratory distress, recurrent upper airway infections and chronic suppurative lung disease. Due to phenotypical similarities with cystic fibrosis (CF) but the relative infrequency of PCD, management of the latter is borrowed from CF protocols for many therapeutic strategies, such as clearance techniques [2]. Unlike for CF, however, the efficacy of such treatments for PCD is less obvious [3, 4]. The explanation likely resides in the different structure and pathophysiology of these entities [5]. Hence, studies to better define the inherent differences between the two conditions might lead to clarify the underlying mechanisms of PCD progression [6].

Assessment of CF and PCD lung disease traditionally includes spirometry and chest computed tomography (CT). Despite the availability of several functional parameters, FEV_1_ still remains a convenient and widely used outcome for both clinical and research purposes. Nevertheless, FEV_1_ deteriorates long after structural damage occurs [7–10], making CT essential in monitoring early and late lung changes in both conditions [1, 11]. However, the perceived risk of ionizing radiation, particularly in the young, limits frequent CT scans. Thus, chest magnetic resonance imaging (MRI) might be a valuable radiation-free alternative [12–14]. Even though its application in pulmonary imaging has long been limited by technical problems such as the low proton density of the lung, increasing evidence supports the reliability of MRI in assessing lung structural damage [12, 13]. Nevertheless, limited access to technology, poor experience in image interpretation, long acquisition times and high costs have prevented chest MRI from being routinely adopted in CF and PCD management.

A recent comparative CT study assessed lung structure in PCD and CF [15] but to our knowledge, no such comparative study using chest MRI has been undertaken to date. The primary aim of the present study was to comparatively assess lung structure in patients with PCD or mild CF by means of chest MRI. The secondary aim was to compare clinical, functional, and microbiological findings in the two cohorts, in order to evaluate different expressions of lung disease.

## Methods

### Study design and patients

This was a prospective, single-center comparative study of PCD and CF lung disease. Between January 2014 and May 2015 we enrolled all mild CF patients, selected on the basis of previously published functional criteria [16], and PCD patients consecutively seen at the Department of Translational Medical Sciences, Federico II University, Naples, who fulfilled the following inclusion criteria: stable lung disease, without acute dyspnea or cough, no pulmonary function changes and no requirements for intravenous antibiotics in the previous 4 weeks [10, 17]. Subjects with acute respiratory infection, developmental delay, or other conditions that could compromise compliance to MRI or spirometry, e.g., age < 6 years and/or claustrophobia, were excluded. No CF patient had undergone neonatal screening, and CF had been diagnosed according to published criteria [18]. Pancreatic insufficiency was defined as stool elastase < 100 μg/g. Abnormal motility and ultrastructural analysis of nasal cilia on transmission electron microscopy confirmed PCD diagnosis [19]. Diagnostic criteria of the enrolled PCD patients were also reviewed according to the recently published international guidelines which require typical ciliary ultrastructure or mutations in PCD causing genes for positive diagnosis, and very low nasal nitric oxide combined with consistent findings at high-speed video microscopy analysis for highly likely diagnosis [20].

At our center, CF patients routinely undergo chest high-resolution CT (HRCT) about every two years [21], whereas the time-interval between consecutive CT scans changes on a case by case basis for PCD. Therefore, when a HRCT was scheduled for routine assessment during follow-up, we presented the study to patients and/or legal guardians, and asked them to undergo chest MRI, spirometry and sputum culture on the same day. We used this approach to verify the reliability of chest MRI scans by real time comparison to CT, the gold standard for lung abnormalities. Clinical data and sputum culture results from the preceding 12 months were collected.

All patients routinely undergo 3-monthly visits. PCD treatment strategy derives from CF care, and includes twice daily chest physiotherapy preceded by nebulized hypertonic saline, and oral and/or intravenous antibiotics based on sputum culture in case of exacerbations. Inhaled antibiotics and/or dornase alpha in PCD are not used in Italy, since they are approved for CF only. Written informed consent was obtained from patients and/or legal guardians. The study was approved by the local Ethical Committee (Comitato Etico per le Attività Biomediche, Federico II University; approval number 184/2014).

### Spirometry

Spirometry was performed according to published criteria [22]. We expressed FVC, FEV_1_, and FEF_25–75_ as Z-scores [23]. We considered FEV_1_ as the primary outcome parameter to assess differences between groups, and a Z-score < −1.64 as abnormal [23].

### Microbiology data

Sputum cultures obtained during the 12 months preceding enrolment were collected. Chronic airway infection was defined when the same pathogen was detected in at least three consecutive cultures within 6 months and after adequate antibiotic therapy.

### MR scanning

MRI was performed with a 3.0-T MR scanner (Magnetom Trio, Siemens Erlangen, Germany), a maximum gradient strength of 40 mT/m, a slew rate of 200 mT/m/ms, and 32 radiofrequency channels. We used a dedicated 12-element integrated matrix coil system that covered the whole thorax for signal reception. It consisted of 1 anterior and 1 posterior flexible phased-array coil, each containing a set of 6 receiver elements. The applied sequence was a T2-weighted half-Fourier single-shot turbo spin-echo (HASTE) sequence, performed using an electrocardiograph-gating to reduce cardiac motion artifacts, and respiratory-gating by a navigator signal that monitored the diaphragm position. The field of view was patient-adapted. Sequence parameters were: repetition time/echo time/flip angle, infinite/92 milliseconds/150 degrees; 25 to 30 slices; slice thickness, 5 mm; distance factor, 20%; transversal orientation (matrix, 380 256); acquisition time, approximately 90 s. Parallel imaging was used for all measurements using the GRAPPA (Generalized Autocalibrating Partially Parallel Acquisition) algorithm with an acceleration factor of 2 and 24 reference lines. No patient required sedation. Door-to-door time was 5.5 min (range, 5–8). All MR studies were of diagnostic quality and were well tolerated.

### HRCT scanning

For all patients CT was part of the routine assessment and did not represent a study procedure. The HRCT scan was performed with a 4-slice CT scanner (Aquilion, Toshiba, Japan) and a bodyweight adapted protocol (adolescents: 120 kV, 140 mAs; children over 45 kg: 120 kV, 65 mAs; children over 35 kg: 120 kV, 45 mAs; children below 35 kg: 120 kV, 30 mAs), with 1x4 mm collimation, 10 mm gap, 0.5 s rotation time, automatic exposure control, multiple inspiratory breath holds of 3 s each, with the patient in a supine position. Scanning extended from the lung apices to below the costophrenic angles. The field of view of each sequence was patient adapted. Images were reconstructed using a high-resolution algorithm. The total time for acquisition of the images was approximately 5 min, including positioning of the patient. Contrast medium was not administered. For documentation of radiation exposure, the dose length product was recorded, and the effective dose (E) and the weighted CT dose index were calculated. A lung window setting (+1500/-500 Hounsfield unit) was used for image analysis. Images were reviewed on a workstation (iMac MacOS 10.4/OsiriX v.2.7.5 32 bit).

### Image evaluation

After removal of identifying information, MRI and CT images, in a randomized patient order, were evaluated to reach consensus between a radiologist and a pediatric pulmonologist with more than 10 years of experience in chest imaging interpretation. In case of disagreement between the two observers, the debated abnormality was scored by the most trained rater. The observers were not directly involved in the patients’ care, and, with the exception of subjects with *situs viscerum inversus* who were easily identifiable as PCD, they were blinded to any clinical and previous radiological data that could bias interpretation. To avoid recall bias, and to prevent raters from being influenced by a previously scored CT while evaluating MR images, CT scans were scored at least 6 weeks after MR images. Further details on image evaluation criteria are reported in the Additional file 1. The scoring system used is detailed in the Additional file 2.

### Statistical analysis

Data are presented as median and ranges, unless otherwise stated. The Mann–Whitney *U* test assessed differences in clinical, functional and structural parameters between groups. Comparisons of functional and structural parameters were reassessed by one-way analysis of covariance to control for diagnostic delay, which was used as covariate. This adjustment allowed to compare lung imaging and lung function data from the two groups undoing the influence of diagnostic delay, which was significantly higher in PCD than in CF. Statistical significance of intragroup comparisons was not determined due to low statistical power deriving from small sample size. Fisher’s exact test was used for categorical variables. Spearman correlation coefficient assessed the relationships among variables. Statistical significance was set at a *p*-value of ≤0.05. Data were analyzed with a statistical software package (SPSS-PC, version 13.0; SPSS; Chicago, IL).

## Results

Thirty-two CF and 28 PCD subjects were eligible. Due to acute respiratory infection 13 subjects (7 CF; 6 PCD) were excluded. Of the remaining, 4 CF and 2 PCD subjects refused MRI due to claustrophobia. One CF patient underwent MRI, but was excluded due to poor compliance resulting in low-quality images. Table 1 summarizes clinical, anthropometric, lung function and microbiological findings from the forty patients ultimately enrolled (20 with PCD, 20 with CF). Cilia ultrastructure of patients with PCD is reported in the Additional file 3. According to the recently published guidelines on PCD diagnosis [20], 16 out of 20 patients met the criteria for a positive PCD diagnosis, due to hallmark ciliary ultrastructure defects. The four remaining patients had a combination of non-typical ciliary defects, very low nasal nitric oxide, and static or circling cilia at the motility study, thus meeting the definition of highly likely PCD diagnosis [20].

**Table 1:** Characteristics of patients with PCD and CF

	PCD	CF	*p*
N	20	20	
Male : Female	12:8	13:7	1
Age (yrs)	15.1 (8.7–29.4)	16 (8–26)	0.60
Age at diagnosis (yrs)	9.9 (0.1–20.5)	0.6 (0–16)	0.03
Age at onset of respiratory symptoms (yrs)	0.1 (0.1–4)	0.6 (0–13)	0.02
Duration of follow-up at tertiary center (yrs)	6.9 (0.1–27.2)	14.5 (0.1–25.9)	0.009
Height (Z-score)	−0.71 (−2.55–1.81)	−0.47 (−2.41–1.5)	0.88
Weight (Z-score)	0.34 (−3.71–2.72)	−0.12 (−3.74–1.43)	0.22
BMI (Z-score)	0.77 (−2.84–2.7)	0.39 (−2.53–1.88)	0.13
Pancreatic insufficiency, n (%)	NA	14 (70)	–
Nasal nitric oxide (ppb)	14 (5–54)	NA	–
*Situs viscerum inversus,* n (%)	12 (60)	NA	–
Systemic antibiotic courses (previous 12 months)	2 (0–7)	2 (0–6)	0.48
Hospital admissions (previous 12 months)	0 (0–1)	0 (0–5)	0.38
FEV_1_ (Z-score)	−1.75 (−4.6–0.7)	−0.9 (−5.4–2.3)	0.24
FVC (Z-score)	−0.6 (−3.9–1.8)	−0.3 (−3.4–2.5)	0.37
FEV_1_/FVC (Z-score)	−1.6 (−3.5–0.1)	−1.1 (−4.2–1.1)	0.24
FEF_25–75_ (Z-score)	−2 (−4.4–0.1)	−1.2 (−5.5–1.1)	0.24
Sputum Microbiology			
Chronic infection by *P. aeruginosa*	1/20	3/20	0.60
Chronic infection by *H. influenzae*	6/20	2/20	0.23
*P. aeruginosa* (≥1 sample)	4/20	11/20	0.05
*H. influenzae* (≥1 sample)	15/20	11/20	0.32
*S. aureus* (≥1 sample)	3/20	13/20	0.003
Genetic analysis			
ΔF508/ΔF508	–	6/20	
ΔF508/other	–	10/20	
other/other	–	4/20	

Data are presented as median and ranges (in parenthesis)

NA not applicable

No differences in age, gender and anthropometric parameters were found between the groups. Pancreatic insufficiency was detected in 70% of CF patients. Despite earlier onset of respiratory symptoms (*p* = 0.02), PCD was diagnosed later than CF, with a delay of approximately 9 years (*p* = 0.03). Duration of follow-up at a tertiary center was significantly longer in CF than PCD (*p* = 0.009). The number of systemic antibiotic courses and hospital admissions during the previous 12 months was comparable in the groups. Similarly, no difference emerged from the comparison between functional parameters. No difference was found between PCD and CF in the proportion of subjects with a FEV_1_ Z-score < −1.64 (50 and 35%, respectively, *p* = 0.5).

Nine PCD (45%) and 15 CF patients (75%) had complete microbiological data, whereas 11 PCD and 5 CF subjects had sputum cultures performed every 4–6 months during the preceding year. Compared to PCD, CF subjects showed a significantly higher prevalence of *Pseudomonas aeruginosa* isolation in at least one sputum sample during the previous year (*p* = 0.05), despite no difference in the prevalence of chronic infection. *Staphylococcus aureus* was more frequently isolated in CF than PCD (*p* = 0.003), whereas no difference was found in the prevalence of both *Haemophilus influenzae* isolation and chronic infection between the groups.

Table 2 summarizes median MRI and CT scores from PCD and CF. For both techniques, total and specific scores were not different, although, both at MRI and CT, severity of collapse/consolidation subscore was higher in PCD than CF (*p* < 0.001). Total scores were slightly lower in pancreatic sufficient *versus* pancreatic insufficient CF at both MRI and CT [8.5 (0–13) *versus* 11.5 (1–15) and 9 (0–13) *versus* 12 (1–15), respectively]. Total MRI and CT scores were not different in PCD *versus* CF (*p* = 0.23 and *p* = 0.21, respectively).

**Table 2:** Chest MRI and CT scores of patients with PCD and CF

	MRI			CT		
	PCD	CF	*p*	PCD	CF	*p*
Severity of bronchiectasis	1.6 ± 0.1	1.5 ± 0.2	0.70	1.7 ± 0.2	1.5 ± 0.2	0.48
Severity of peribronchial wall thickening	1.7 ± 0.1	1.4 ± 0.2	0.09	1.8 ± 0.09	1.5 ± 0.1	0.14
Extent of bronchiectasis	1.9 ± 0.2	2 ± 0.2	0.49	1.9 ± 0.2	2 ± 0.2	0.58
Extent of mucous plugging	1.6 ± 0.2	1.2 ± 0.2	0.28	1.7 ± 0.2	1.3 ± 0.2	0.36
Extent of sacculations or abscesses	0.05 ± 0.05	0	0.33	0.05 ± 0.05	0	0.33
Generation of bronchial divisions involved (bronchiectasis or plugging)	2.9 ± 0.1	2.3 ± 0.2	0.07	2.9 ± 0.1	2.3 ± 0.2	0.07
Severity of bullae	0	0	–	0.2 ± 0.2	0	0.16
Severity of emphysema	0.1 ± 0.07	0	0.16	0.1 ± 0.07	0	0.16
Severity of collapse or consolidations	1.6 ± 0.1	0.6 ± 0.2	<0.001	1.6 ± 0.1	0.6 ± 0.2	<0.001
Total score	11.6 ± 0.7	9.1 ± 1	0.23	12 ± 0.8	9.3 ± 1	0.21

Data are presented as mean and standard error of the mean

In PCD and CF, total MRI score was in the mild range (0–9) in 15 and 45%, and in the moderate range (10–18) in 85 and 55% of cases, respectively (*p* > 0.05 for both comparisons).

**Fig. 1:**
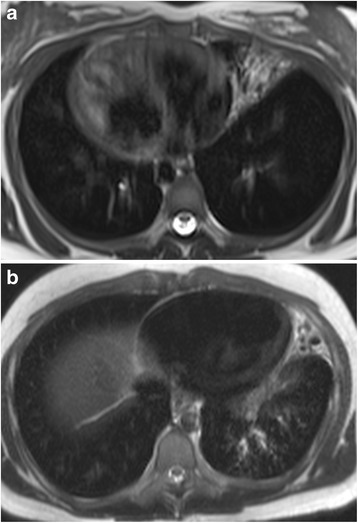
Transversal MR images of a 14–year-old girl with PCD and *situs viscerum inversus* showing an area of consolidation in the middle lobe (**a**), and of a 15–year-old boy with CF showing an area of consolidation in the lingula and sparse bronchiectasis in the left lower lobe (**b**)

Image evaluation in PCD and CF showed excellent agreement between the techniques for all scores (r > 0.9). We could not compute agreement between CT and MRI scores for extent of CF sacculations/abscesses, severity of bullae and severity of emphysema, because of the constant value of these categories at CT and MRI in all subjects. Similarly, no agreement was calculated for MRI severity of PCD bullae due to the constant value of this category. Only severity of emphysema showed poor agreement (*r* = 0.44) between PCD CT and MRI score. Figs. 1a and b are examples of chest MRI from a PCD and a CF patient, respectively.

Total MRI score was significantly related to FEV_1_ Z-score in PCD (*r* = −0.45, *p* = 0.04) and CF (*r* = −0.43, *p* = 0.05).

Due to relevant difference in age at diagnosis between PCD and CF, we comparatively re-assessed lung function and structure after control for diagnostic delay. We confirmed our original findings, as no differences emerged between PCD and CF for spirometry and MRI or CT scores (Table 3). The higher score for severity of collapse/consolidation in PCD was confirmed after correction for diagnostic delay.

**Table 3:** Lung imaging and lung function of PCD and CF patients controlled for diagnostic delay

	MRI			CT		
	PCD	CF	*p*	PCD	CF	*p*
Severity of bronchiectasis	1.6 ± 0.2	1.6 ± 0.2	0.9	1.7 ± 0.2	1.6 ± 0.2	0.9
Severity of peribronchial wall thickening	1.7 ± 0.2	1.5 ± 0.2	0.4	1.8 ± 0.1	1.7 ± 0.1	0.4
Extent of bronchiectasis	1.8 ± 0.2	2.2 ± 0.2	0.3	1.8 ± 0.2	2.3 ± 0.2	0.3
Extent of mucous plugging	1.6 ± 0.2	1.3 ± 0.3	0.4	1.6 ± 0.3	1.5 ± 0.3	0.5
Extent of sacculations or abscesses	0.02 ± 0.04	0.03 ± 0.04	0.2	0.02 ± 0.04	0.03 ± 0.04	0.2
Generation of bronchial divisions involved (bronchiectasis or plugging)	2.8 ± 0.2	2.7 ± 0.2	0.1	2.8 ± 0.2	2.6 ± 0.2	0.1
Severity of bullae	0	0	–	0.1 ± 0.1	0.1 ± 0.1	0.1
Severity of emphysema	0.08 ± 0.05	0.03 ± 0.06	0.2	0.06 ± 0.05	0.04 ± 0.06	0.1
Severity of collapse or consolidations	1.5 ± 0.2	0.8 ± 0.2	<0.001	1.5 ± 0.2	0.8 ± 0.2	<0.001
Total score	11.0 ± 0.9	10.1 ± 0.9	0.1	11.4 ± 0.9	10.4 ± 0.9	0.1
Lung function	PCD		CF		*p*	
FVC (Z-score)	−0.3 ± 0.3		−0.5 ± 0.3		0.08	
FEV_1_ (Z-score)	−1.4 ± 0.4		−1.4 ± 0.4		0.1	
FEF_25–75_ (Z-score)	−2.0 ± 0.4		−1.8 ± 0.4		0.4	
FEV_1_/FVC (Z-score)	−1.7 ± 0.3		−1.4 ± 0.3		0.5	

Data are presented as mean and standard error of the mean

## Discussion

Recently CF and PCD have been increasingly compared [15, 24–29]. To our knowledge, this is the first study that comparatively assessed PCD and CF lung disease using MRI. Our main finding is the absence of striking differences in lung function and structure between the two cohorts. Actually, we found a significant difference between patients’ age at PCD or CF diagnosis, hence follow-up was longer in CF. We initially guessed that PCD deteriorated as CF because diagnosis occurred late and patients were referred to a tertiary care center after longer periods of inadequate management. Surprisingly, once controlled for diagnostic delay, the absence of any difference between the groups was confirmed, with the only exception of the collapse/consolidation subscore that was persistently more severe in PCD. Overall, these data add further evidence to the widespread concept that PCD is not the mild disease believed.

The relevant delay in the ultimate diagnosis is universally recognized in PCD [1, 30], and we confirm it. What additionally emerges from current data is that a delayed diagnosis is not the sole responsible for the similarities found, hence further explanation should be provided. Despite counterintuitive, the lack of association between early PCD diagnosis and better pulmonary outcomes, strongly suggested by our results, has been highlighted previously [31, 32]. Furthermore, a recent longitudinal study of adult PCD subjects has confirmed that, even though negatively associated with baseline FEV_1_, age at diagnosis does not correlate with longitudinal functional measurements, thus raising doubts on the real impact of optimal management at an early stage on the subsequent disease progression [33]. These studies did not correlate chest CT scores with age at diagnosis, but it is reasonable to hypothesize that, likewise functional impairment, also structural abnormalities are poorly affected by it. Nevertheless, the controversy surrounding the association between age at PCD diagnosis and lung function impairment clearly brings into focus the need for future larger studies.

It is well known that in CF chronic depletion of periciliary layer volume results in hyperviscous mucus layer adhesion to cells, thus inhibiting cough clearance [34]. Conversely, the PCD periciliary layer volume is apparently normal and airway surface well hydrated, hence cough-dependent mucus clearance is preserved [35]. However, despite distinct basic defects [19, 36], CF and PCD share a common hallmark, i.e., retention of airways mucus and growth of biofilms [5, 37]. Although we do not provide data on therapeutic adherence, the poorer PCD patients’ compliance to treatment in our practice might explain the similarities we found, despite comparable time interval between visits. In PCD poor compliance to treatment likely derives from unawareness of potential threats of the disease, especially if inadequately treated [38]. Conversely, CF patients, particularly those with good nutritional status like ours, generally show adequate prescriptions adherence [39]. Inhaled antibiotics and dornase alpha, which in Italy are authorized only for CF, are not routinely used [40], and this might help interpreting our findings. Future comparative studies might realize novel PCD management strategies, and the efficacy of drugs not routinely used in PCD should be further investigated. A final, critical point is that the genetic background may influence PCD lung disease expression [41]. As we cannot provide PCD genotype, a selection bias is not excluded.

Few original studies have compared PCD and CF lung disease [15, 25]. A comparative analysis of chest CT, also including PCD patients from our center, reported less lung changes in PCD than CF [25]. In that study, PCD patients were compared to a previously published CF cohort undergoing chest imaging for clinical reasons, unlike current CF subjects [42]. The difference between imaging scoring systems makes any comparison between the two studies unreliable. A recent study found that lung disease severity at CT was similar between PCD and pancreatic sufficient CF, but significantly higher in pancreatic insufficient CF [15]. Conversely, in the current study we did not find differences between the two diseases in terms of lung function and structure, except collapse/consolidation. Comparing that study population with ours, it is worth noting that age at enrolment was similar in PCD and CF. Nevertheless, our CF patients had milder pulmonary involvement, with fewer exacerbations and less chronic *Pseudomonas aeruginosa* infection rate (15% *versus* 51%, respectively) than the comparison cohort. More importantly, in the current study lung damage at MRI was in the moderate range in approximately half of CF *versus* two thirds of PCD, with the remaining subjects from both groups showing mild impairment. Actually, FEV_1_ Z-score was normal in our CF and abnormal in our PCD subjects (−0.9 *versus −*1.75, respectively), although the difference was not significant. Overall, these data suggest that the CF patients from the two studies differ in disease severity, despite comparable lung function, with milder pulmonary disease in our cohort [15]. Unfortunately, the small number of our CF patients with or without pancreatic insufficiency precluded the comparison between these CF subgroups. Furthermore, the limited sample size may have biased our findings.

Our study has both strengths and limitations. Undoubtedly the use of a non-invasive tool, namely MRI, is an element of novelty in the comparison of CF and PCD. Chest MRI has been used in both diseases separately, and its reliability has been widely demonstrated through comparisons with traditional techniques [12, 13]. We found an excellent agreement between MRI and CT for most abnormality parameters in both PCD and CF. However, although CT is a useful staging test, it is impractical for monitoring lung disease because of radiation burden [43]. Conversely, spirometry is an insensitive marker of lung disease progression [10, 42]. In this setting, MRI is attractive and reliable as radiation-free option [13, 44, 45]. In addition, the re-analysis of data after control for diagnostic delay highlighted that the lack of differences between PCD and CF is unlikely due to prolonged untreated PCD.

Our study has also limitations. First, as shown by chest imaging scores and spirometry, our analysis compared mild CF subjects and PCD patients mostly presenting mild-to-moderate functional and structural impairment, with virtually no patients showing more severe disease. Moreover, the modified Helbich score does not take into account the non-comparable involvement of different lobes. However, we opted for this scoring system as it was previously used in CF and PCD, and because the observers were already trained in it. In addition, as previously stated, enrolled CF patients presented mild lung disease, unlike PCD subjects whose pulmonary impairment was likely more severe. Indeed, despite CF and PCD recruitment was timed to coincide with a routine CT, the lack of a shared protocol defining the time interval between scans in PCD might have determined the enrolment of more severe PCD. Similarly, it could also be speculated that the enrolled PCD patients have more severe disease entailing referral compared to those with a late or totally missed diagnosis, and this could further bias results. Indeed, the majority of our PCD patients fully met the stringent definition for positive PCD diagnosis recently formalized by international guidelines and only in four of them findings from several diagnostic tests made PCD diagnosis “highly likely”. For these subjects genetic testing is certainly warranted, but given the highly suggestive clinical picture, the very low nasal nitric oxide levels, the abnormal cilia motility and the ultrastructural defects found – even though not hallmark of PCD – we felt they could be included in our PCD cohort. Of course, we deeply commend the ERS Task Force for the effort in standardizing the diagnostic pathway in PCD. Their guidelines, requiring an interaction between more diagnostic tools to achieve a definite diagnosis, will certainly help in the characterization of patients and in correlating disease severity with cilia ultrastructure and motility, and with the genetic background. This will also strengthen data from multicenter PCD studies enrolling patients whose diagnosis will no longer be questioned.

The mentioned drawbacks, together with the limited sample size, make it difficult to generalize the data. Further research on larger populations from multicenter sites, possibly including all ranges of severity is needed to verify whether our results are replicable. Finally, longitudinal studies comparing PCD and CF from early life to adolescence/adulthood would likely improve our knowledge on differences in the speed of lung disease progression between the two entities.

## Conclusions

This comparative study of PCD and CF suggests that the two conditions may share similar lung function and MRI changes and confirms that chest MRI is a valuable radiation-free tool. Comparative studies of PCD and CF lung disease may hopefully also help to develop PCD-specific protocols not derived from CF.

## Additional files

Additional file 1: Image evaluation. (DOC 24 kb)

Additional file 2: Modified Helbich scoring system for HRCT and MRI. (DOC 39 kb)

Additional file 3: *Situs viscerum inversus* and cilia ultrastructure of patients with PCD. (DOC 31 kb)

## Abbreviations

CF: Cystic fibrosis
CT: Computed tomography
FEF_25–75_: Forced expiratory flow between 25% and 75% of FVC
FEV_1_: Forced expiratory volume in the first second
FVC: Forced vital capacity
HRCT: High-resolution computed tomography
MRI: Magnetic resonance imaging
PCD: Primary ciliary dyskinesia

## References

[1] Lucas JS, Burgess A, Mitchison HM, Moya E, Williamson M, Hogg C, National PCD, Service UK (2014). Diagnosis and management of primary ciliary dyskinesia. Arch Dis Child.

[2] Shapiro AJ, Zariwala MA, Ferkol T, Davis SD, Sagel SD, Dell SD, Genetic Disorders of Mucociliary Clearance Consortium (2016). Diagnosis, monitoring, and treatment of primary ciliary dyskinesia: PCD foundation consensus recommendations based on state of the art review. Pediatr Pulmonol.

[3] Desai M, Weller PH, Spencer DA (1995). Clinical benefit from nebulized human recombinant DNase in Kartagener’s syndrome. Pediatr Pulmonol.

[4] Wills PJ, Wodehouse T, Corkery K, Mallon K, Wilson R, Cole PJ (1996). Short-term recombinant human DNase in bronchiectasis. Effect on clinical state and in vitro sputum transportability. Am J Respir Crit Care Med.

[5] Knowles MR, Boucher RC (2002). Mucus clearance as a primary innate defense mechanism for mammalian airways. J Clin Invest.

[6] Polineni D, Davis SD, Dell SD (2016). Treatment recommendations in Primary Ciliary Dyskinesia. Paediatr Respir Rev.

[7] Pellegrino R, Viegi G, Brusasco V, Crapo RO, Burgos F, Casaburi R (2005). Interpretative strategies for lung function tests. Eur Respir J.

[8] Santamaria F, Grillo G, Guidi G, Rotondo A, Raia V, de Ritis G (1998). Cystic fibrosis: when should high-resolution computed tomography of the chest Be obtained?. Pediatrics.

[9] Santamaria F, Montella S, Camera L, Palumbo C, Greco L, Boner AL (2006). Lung structure abnormalities, but normal lung function in pediatric bronchiectasis. Chest.

[10] Maglione M, Bush A, Montella S, Mollica C, Manna A, Esposito A (2012). Progression of lung disease in primary ciliary dyskinesia: is spirometry less accurate than CT?. Pediatr Pulmonol.

[11] Tiddens HA, Rosenow T (2014). What did we learn from two decades of chest computed tomography in cystic fibrosis?. Pediatr Radiol.

[12] Puderbach M, Eichinger M, Gahr J, Ley S, Tuengerthal S, Schmähl A (2007). Proton MRI appearance of cystic fibrosis: comparison to CT. Eur Radiol.

[13] Montella S, Santamaria F, Salvatore M, Pignata C, Maglione M, Iacotucci P (2009). Assessment of chest high-field magnetic resonance imaging in children and young adults with noncystic fibrosis chronic lung disease: comparison to high-resolution computed tomography and correlation with pulmonary function. Invest Radiol.

[14] Montella S, Mollica C, Finocchi A, Pession A, Pietrogrande MC, Trizzino A (2013). Non invasive assessment of lung disease in ataxia telangiectasia by high-field magnetic resonance imaging. J Clin Immunol.

[15] Cohen-Cymberknoh M, Simanovsky N, Hiller N, Gileles Hillel A, Shoseyov D, Kerem E (2014). Differences in disease expression between primary ciliary dyskinesia and cystic fibrosis with and without pancreatic insufficiency. Chest.

[16] Schluchter MD, Konstan MW, Drumm ML, Yankaskas JR, Knowles MR (2006). Classifying severity of cystic fibrosis lung disease using longitudinal pulmonary function data. Am J Respir Crit Care Med.

[17] Soni R, Dobbin CJ, Milross MA, Young IH, Bye PP (2008). Gas exchange in stable patients with moderate-to-severe lung disease from cystic fibrosis. J Cyst Fibros.

[18] Rosenstein BJ, Cutting GR (1998). The diagnosis of cystic fibrosis: a consensus statement. Cystic Fibrosis Foundation Consensus Panel. J Pediatr.

[19] Bush A, Hogg C (2012). Primary ciliary dyskinesia: recent advances in epidemiology, diagnosis, management and relationship with the expanding spectrum of ciliopathy. Expert Rev Respir Med.

[20] Lucas JS, Barbato A, Collins SA, Goutaki M, Behan L, Caudri D (2017). European Respiratory Society guidelines for the diagnosis of primary ciliary dyskinesia. Eur Respir J.

[21] Tiddens HA, Stick SM, Davis S (2014). Multi-modality monitoring of cystic fibrosis lung disease: the role of chest computed tomography. Paediatr Respir Rev.

[22] Miller MR, Hankinson J, Brusasco V, Burgos F, Casaburi R, Coates A, ATS/ERS Task Force (2005). Standardisation of spirometry. Eur Respir J.

[23] Quanjer PH, Stanojevic S, Cole TJ, Baur X, Hall GL, Culver BH, ERS Global Lung Function Initiative (2012). Multi-ethnic reference values for spirometry for the 3-95-yr age range: the global lung function 2012 equations. Eur Respir J.

[24] Horváth I, Loukides S, Wodehouse T, Csiszér E, Cole PJ, Kharitonov SA (2003). Comparison of exhaled and nasal nitric oxide and exhaled carbon monoxide levels in bronchiectatic patients with and without primary ciliary dyskinesia. Thorax.

[25] Santamaria F, Montella S, Tiddens HA, Guidi G, Casotti V, Maglione M (2008). Structural and functional lung disease in primary ciliary dyskinesia. Chest.

[26] Mackerness KJ, Jose PJ, Bush A (2009). Differences in airway inflammation in cystic fibrosis and primary ciliary dyskinesia. Pediatr Asthma Allergy Immunol.

[27] Irving SJ, Ives A, Davies G, Donovan J, Edey AJ, Gill SS (2013). Lung clearance index and high-resolution computed tomography scores in primary ciliary dyskinesia. Am J Respir Crit Care Med.

[28] Montuschi P, Paris D, Montella S, Melck D, Mirra V, Santini G (2014). Nuclear magnetic resonance-based metabolomics discriminates primary ciliary dyskinesia from cystic fibrosis. Am J Respir Crit Care Med.

[29] Joensen O, Paff T, Haarman EG, Skovgaard IM, Jensen PØ, Bjarnsholt T (2014). Exhaled breath analysis using electronic nose in cystic fibrosis and primary ciliary dyskinesia patients with chronic pulmonary infections. PLoS One.

[30] Kuehni CE, Frischer T, Strippoli MP, Maurer E, Bush A, Nielsen KG, ERS Task Force on Primary Ciliary Dyskinesia in Children (2010). Factors influencing age at diagnosis of primary ciliary dyskinesia in European children. Eur Respir J.

[31] Marthin JK, Petersen N, Skovgaard LT, Nielsen KG (2010). Lung function in patients with primary ciliary dyskinesia: a cross-sectional and 3-decade longitudinal study. Am J Respir Crit Care Med.

[32] Maglione M, Bush A, Nielsen KG, Hogg C, Montella S, Marthin JK (2014). Multicenter analysis of body mass index, lung function, and sputum microbiology in primary ciliary dyskinesia. Pediatr Pulmonol.

[33] Shah A, Shoemark A, MacNeill SJ, Bhaludin B, Rogers A, Bilton D (2016). A longitudinal study characterising a large adult primary ciliary dyskinesia population. Eur Respir J.

[34] Itani OA, Chen JH, Karp PH, Ernst S, Keshavjee S, Parekh K (2011). Human cystic fibrosis airway epithelia have reduced Cl- conductance but not increased Na + conductance. Proc Natl Acad Sci U S A.

[35] Livraghi A, Randell SH (2007). Cystic fibrosis and other respiratory diseases of impaired mucus clearance. Toxicol Pathol.

[36] Rowe SM, Miller S, Sorscher EJ (2005). Cystic fibrosis. N Engl J Med.

[37] Bush A, Payne D, Pike S, Jenkins G, Henke MO, Rubin BK (2006). Mucus properties in children with primary ciliary dyskinesia: comparison with cystic fibrosis. Chest.

[38] Pifferi M, Bush A, Di Cicco M, Pradal U, Ragazzo V, Macchia P (2010). Health-related quality of life and unmet needs in patients with primary ciliary dyskinesia. Eur Respir J.

[39] Shakkottai A, Kidwell KM, Townsend M, Nasr SZ (2015). A five-year retrospective analysis of adherence in cystic fibrosis. Pediatr Pulmonol.

[40] Strippoli MP, Frischer T, Barbato A, Snijders D, Maurer E, Lucas JS, ERS Task Force on Primary Ciliary Dyskinesia in Children (2012). Management of primary ciliary dyskinesia in European children: recommendations and clinical practice. Eur Respir J.

[41] Davis SD, Ferkol TW, Rosenfeld M, Lee HS, Dell SD, Sagel SD (2015). Clinical features of childhood primary ciliary dyskinesia by genotype and ultrastructural phenotype. Am J Respir Crit Care Med.

[42] de Jong PA, Lindblad A, Rubin L, Hop WC, de Jongste JC, Brink M (2006). Progression of lung disease on computed tomography and pulmonary function tests in children and adults with cystic fibrosis. Thorax.

[43] Brenner DJ, Hall EJ (2007). Computed tomography--an increasing source of radiation exposure. N Engl J Med.

[44] Montella S, Maglione M, Bruzzese D, Mollica C, Pignata C, Aloj G (2012). Magnetic resonance imaging is an accurate and reliable method to evaluate non-cystic fibrosis paediatric lung disease. Respirology.

[45] Owrangi AM, Parraga G (2012). Chest MRI in children: why bother?. Respirology.

