# Supplementary material for: Lung structure and function similarities between primary ciliary dyskinesia and mild cystic fibrosis: a pilot study
Source: Ital J Pediatr. 2017 Apr 12;43:34. doi: 10.1186/s13052-017-0351-2 (PMC5389053; doi:10.1186/s13052-017-0351-2)
Supplement: Supplementary file 1 — Image evaluation. (DOC 24 kb) [file 13052_2017_351_MOESM1_ESM.doc]

**Additional File 1**

*Image Evaluation*

We scored both CT and MR scans using the morphologic scoring system which was originally developed for CF by Helbich et al. [1], later modified by Puderbach et al. [2], and recently used also for the assessment of PCD lung disease [3] (Additional file 2). Maximum achievable total score was 25, indicating the most severe lung changes. For the purpose of quantifying the severity of PCD or CF lung structure deterioration, we arbitrarily divided the total MR score into three scores subgroups, i.e. 0-9, or mild; 10-18, or moderate; and 19-25, or severe lung damage, respectively.

For the analysis of the specific CT and MR parameters included in the scoring system we referred to accepted definitions described in detail elsewhere [2]. Furthermore, for the categories “severity of bronchiectasis” and “severity of peribronchial wall thickening”, we recorded the most prevalent degree of severity. It was not possible to assess peribronchial wall thickening in the presence of mucous plugging, and, if mucous plugging was seen within the periphery of a lung segment, bronchiectasis was scored also in that segment. Six lobes were examined, the lingula being scored as a separate lobe. In patients with *situs viscerum inversus*, the right lung was the lung in which the middle lobar bronchus and the corresponding middle lobe were identified at scans.

**References**

1. Helbich TH, Heinz-Peer G, Eichler I, Wunderbaldinger P, Götz M, Wojnarowski C, et al. Cystic fibrosis: CT assessment of lung involvement in children and adults. Radiology. 1999;213:537-44.

2. Puderbach M, Eichinger M, Haeselbarth J, Ley S, Kopp-Schneider A, Tuengerthal S, et al. Assessment of morphological MRI for pulmonary changes in cystic fibrosis (CF) patients: comparison to thin-section CT and chest x-ray. Invest Radiol. 2007;42:715-25.

3. Montella S, Santamaria F, Salvatore M, Pignata C, Maglione M, Iacotucci P, et al. Assessment of chest high-field magnetic resonance imaging in children and young adults with noncystic fibrosis chronic lung disease: comparison to high-resolution computed tomography and correlation with pulmonary function. Invest Radiol. 2009;44:532-8.
