# Supplementary material for: Lung structure and function similarities between primary ciliary dyskinesia and mild cystic fibrosis: a pilot study
Source: Ital J Pediatr. 2017 Apr 12;43:34. doi: 10.1186/s13052-017-0351-2 (PMC5389053; doi:10.1186/s13052-017-0351-2)
Supplement: Supplementary file 2 — Modified Helbich scoring system for HRCT and MRI. (DOC 39 kb) [file 13052_2017_351_MOESM2_ESM.doc]

**Additional File 2.** Modified Helbich scoring system for HRCT and MRI.

|  | **Score** | | | |
| --- | --- | --- | --- | --- |
| **Category** | **0** | **1** | **2** | **3** |
| Severity of bronchiectasis | Absent | Mild (lumen slightly greater than the diameter of adjacent vessel) | Moderate (lume 2 to 3 times the diameter of the vessel) | Severe (lumen >3 times the diameter of the vessel) |
| Severity of peribronchial wall thickening | Absent | Mild (wall thickness equal to the diameter of adjacent vessel) | Moderate (wall thickness greater than and up to twice the diameter of adjacent vessel) | Severe (wall thickness more than twice the diameter of adjacent vessel) |
| Extent of bronchiectasis | Absent | 1-5^#^ | 6-9^#^ | >9^#^ |
| Extent of mucous plugging | Absent | 1-5^#^ | 6-9^#^ | >9^#^ |
| Extent sacculations or abscesses | Absent | 1-5^#^ | 6-9^#^ | >9^#^ |
| Generation of bronchial divisions involved (bronchiectasis or plugging) | Absent | Up to the 4^th^ generation | Up to the 5^th^ generation | Up to the 6^th^ generation and distal |
| Severity of bullae | Absent | Unilateral (not >4) | Bilateral (not >4) | >4 |
| Severity of emphysema | Absent | 1-5^#^ | >5^#^ | Not applicable |
| Severity of collapse or consolidation | Absent | Subsegmental | Segmental or lobar | Not applicable |

^#^ Number of bronchopulmonary segments.
