# Supplementary material for: Lung structure and function similarities between primary ciliary dyskinesia and mild cystic fibrosis: a pilot study
Source: Ital J Pediatr. 2017 Apr 12;43:34. doi: 10.1186/s13052-017-0351-2 (PMC5389053; doi:10.1186/s13052-017-0351-2)
Supplement: Supplementary file 3 — Situs viscerum inversus and cilia ultrastructure of patients with PCD. (DOC 31 kb) [file 13052_2017_351_MOESM3_ESM.doc]

| **Additional File 3.** *Situs viscerum inversus* and cilia ultrastructure of patients with PCD. | |
| --- | --- |
|  | **PCD patients** |
| n | 20 |
| *Situs viscerum inversus*, n (%) | 12 (60) |
| Cilia ultrastructure, n (%) |  |
| Outer or combined outer and inner dynein arms absence | 15 (75) |
| Isolated inner dynein arm absence | 2 (10) |
| Isolated axonemal disorganization | 2 (10) |
| Axonemal disorganization and inner dynein arm absence | 1 (5) |
|  |  |
